# A candidate gene study of capecitabine-related toxicity in colorectal cancer identifies new toxicity variants at *DPYD* and a putative role for *ENOSF1* rather than *TYMS*

**DOI:** 10.1136/gutjnl-2013-306571

**Published:** 2014-03-19

**Authors:** Dan Rosmarin, Claire Palles, Alistair Pagnamenta, Kulvinder Kaur, Guillermo Pita, Miguel Martin, Enric Domingo, Angela Jones, Kimberley Howarth, Luke Freeman-Mills, Elaine Johnstone, Haitao Wang, Sharon Love, Claire Scudder, Patrick Julier, Ceres Fernández-Rozadilla, Clara Ruiz-Ponte, Angel Carracedo, Sergi Castellvi-Bel, Antoni Castells, Anna Gonzalez-Neira, Jenny Taylor, Rachel Kerr, David Kerr, Ian Tomlinson

**Affiliations:** 1 Molecular and Population Genetics Laboratory, Oxford, UK; 2 Department of Oncology, University of Oxford, Old Road Campus Research Building, Oxford, UK; 3 Oxford NIHR Comprehensive Biomedical Research Centre, Wellcome Trust Centre for Human Genetics, Oxford, UK; 4 Human Genotyping Unit-CeGen, Human Cancer Genetics Programme, Spanish National Cancer Research Centre, Melchor Fernández Almagro 3, Madrid, Spain; 5 Department of Medical Oncology, Instituto de Investigacion Sanitaria Hospital General Universitario Gregorio Marañón, Universidad Complutense, Madrid, Spain; 6 Centre for Statistics in Medicine, University of Oxford, Botnar Research Centre, Oxford, UK; 7 OCTO, University of Oxford, Old Road Campus Research Building, Oxford, UK; 8 Galician Public Foundation of Genomic Medicine (FPGMX), CIBERER, Genomics Medicine Group, Hospital Clinico, University of Santiago de Compostela, Santiago de Compostela, Galicia, Spain; 9 Genetic Susceptibility to Colorectal Cancer Group, Gastrointestinal & Pancreatic Oncology Team, IDIBAPS/CIBERehd/Hospital Clínic, Centre Esther Koplowitz (CEK), Barcelona, Spain; 10 Institute of Digestive and Metabolic Diseases, Hospital Clínic, Barcelona, Spain; 11 Nuffield Department of Clinical Laboratory Sciences, University of Oxford, Oxford, UK

**Keywords:** Cancer, Cancer Genetics, Chemotherapy, Pharmacogenetics

## Abstract

**Objective:**

Capecitabine is an oral 5-fluorouracil (5-FU) pro-drug commonly used to treat colorectal carcinoma and other tumours. About 35% of patients experience dose-limiting toxicity. The few proven genetic biomarkers of 5-FU toxicity are rare variants and polymorphisms, respectively, at candidate loci dihydropyrimidine dehydrogenase (*DPYD*) and thymidylate synthase (*TYMS*).

**Design:**

We investigated 1456 polymorphisms and rare coding variants near 25 candidate 5-FU pathway genes in 968 UK patients from the QUASAR2 clinical trial.

**Results:**

We identified the first common *DPYD* polymorphisms to be consistently associated with capecitabine toxicity, rs12132152 (toxicity allele frequency (TAF)=0.031, OR=3.83, p=4.31×10^−6^) and rs12022243 (TAF=0.196, OR=1.69, p=2.55×10^−5^). rs12132152 was particularly strongly associated with hand-foot syndrome (OR=6.1, p=3.6×10^−8^). The rs12132152 and rs12022243 associations were independent of each other and of previously reported *DPYD* toxicity variants*.* Next-generation sequencing additionally identified rare *DPYD* variant p.Ala551Thr in one patient with severe toxicity. Using functional predictions and published data, we assigned p.Ala551Thr as causal for toxicity. We found that polymorphism rs2612091, which lies within an intron of *ENOSF1,* was also associated with capecitabine toxicity (TAF=0.532, OR=1.59, p=5.28×10^−6^). *ENSOF1* is adjacent to *TYMS* and there is a poorly characterised regulatory interaction between the two genes/proteins. Unexpectedly, rs2612091 fully explained the previously reported associations between capecitabine toxicity and the supposedly functional *TYMS* variants, 5′VNTR 2R/3R and 3′UTR 6 bp ins-del. rs2612091 genotypes were, moreover, consistently associated with *ENOSF1* mRNA levels, but not with *TYMS* expression.

**Conclusions:**

*DPYD* harbours rare and common capecitabine toxicity variants. The toxicity polymorphism in the *TYMS* region may actually act through *ENOSF1*.

**Significance of this study**

**What is already known on this subject?**

- Chemotherapy based on 5-fluorouracil (5-FU) is used for the treatment of several common types of cancer. For colorectal carcinoma, the 5-FU pro-drug capecitabine is often given routinely after surgery for stage II or III tumours.
- The benefits of 5-FU are modest (<5% increased long-term survival) and must be weighed against drug toxicity. Inherited variation in genes involved in 5-FU metabolism can explain some of this toxicity.
- However, only four genetic variants in the 5-FU pathway have good prior evidence of association with toxicity.

**What are the new findings?**

- We found four new associations between genetic variants and capecitabine toxicity.
- Three of these associations involved variants, two common and one rare, at the dihydropyrimidine dehydrogenase (*DPYD*) locus.
- The fourth association involved a polymorphism within *ENOSF1,* a gene adjacent to thymidylate synthase (*TYMS*).
- Statistically, the ENOSF1 association completely explains two previously reported 5-FU toxicity polymorphisms (5′VNTR and 3′UTR) in the thymidylate synthase gene.
- Most patients with severe myelosuppression carried a rare *DPYD* allele: *2A, 2846T>A, *13 or A551T.

**How might it impact on clinical practice in the foreseeable future?**

- The genetic architecture of 5-FU/capecitabine toxicity is complex and encompasses rare and common variants.
- Panels of markers in tests used to predict clinically actionable 5-FU/capecitabine toxicity should be updated to include the new *DPYD* and *ENOSF1* alleles, while omitting the *TYMS* 5′VNTR and 3′UTR polymorphisms.
- Rare loss-of-function *DPYD* alleles remain the only genetic variants proven to have large positive predictive values for 5-FU/capecitabine toxicity, and these variants account for the majority of patients with life-threatening myelosuppression induced by capecitabine.

## Introduction

Capecitabine (Xeloda, Roche) is an oral 5-fluorouracil (5-FU) pro-drug commonly given to patients with colorectal cancer (CRC). Capecitabine is activated to 5-FU, which then causes cytotoxicity by inhibiting production of thymidine and by being converted to metabolites that are incorporated into DNA and RNA.^1^ As with other 5-FU-based chemotherapy regimens, approximately one-third of capecitabine patients suffer dose-limiting levels of drug-induced adverse events. The rapid onset of toxicity results in mortality for 0.5–2% of patients in monotherapy and combination regimens of infusional and bolus 5-FU,^2^ and about half that number for capecitabine schedules.

The most common dose-limiting capecitabine toxicities are hand-foot syndrome (HFS) and diarrhoea. Additionally, an important proportion of patients develop neutropaenia and thromboctytopaenia, and others experience nausea, vomiting, mucositis and stomatitis. Some interpatient differences in toxicity can be explained by clinical factors, such as age, gender, local clinical practice and, possibly, diet.^3–5^ However, much variability in toxicity remains unexplained.

**Figure 1:**
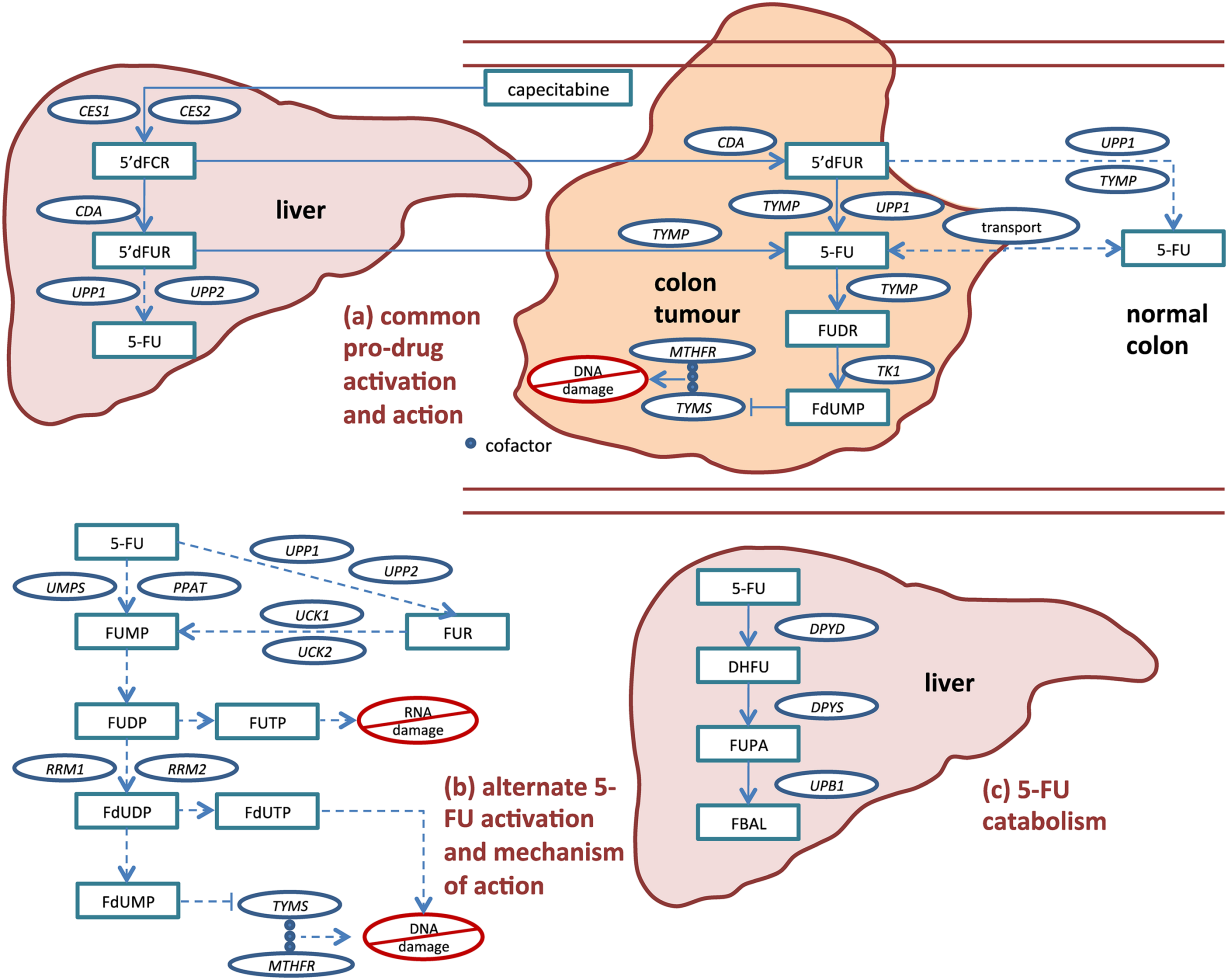
Capecitabine activation and subsequent 5-FU activation, action, transport and catabolism. Capecitabine is an oral 5-FU prodrug that is rationally designed so that concentrations of the cytotoxic metabolites FdUMP, FdUTP and FUTP are higher within malignant cells than within normal cells. After absorption in the gut, most of the drug activation occurs via the common pro-drug activation route (a), starting in the liver and finishing in the tumour. Additionally, 5-FU can be converted to its active compounds via alternate activation routes (b) in colon tumour cells and cells from multiple other tissues. Toxicity may occur if non-target tissue is exposed to activated capecitabine/5-FU (ie, FdUMP, FdUTP and FUTP), either when 5-FU is activated prior to arrival at the tumour or when it exits target tissue and is subsequently activated. 5-FU released in circulation may be quickly metabolised (c) in the liver by enzymes including DPYD. Gene names for candidate enzymes, provided in circles, are defined in online supplementary table S1. Drug catabolites are provided in rectangles. Primary (rationally designed) pathway are shown in solid lines, and alternative pathways shown in dashed lines.

The biochemical pathway of capecitabine activation and subsequent 5-FU action and degradation is well established and provides 25 candidate genes in which variation might affect 5-FU toxicity (figure 1, and see online supplementary table 1).^6^ Upon absorption in the gut, capecitabine is partially converted to 5-FU in the liver, then preferentially converted to 5-FU at the CRC site. Much 5-FU is degraded in the liver by dihydropyrimidine dehydrogenase (DPYD) prior to activation. As part of the drug's rationally designed activation, 5-FU is further activated in the tumour to cytotoxic compounds that inhibit DNA synthesis by competing with nucleotide precursors for binding with thymidylate synthase (TYMS). Various sources of toxicity may exist, including alternative activation pathways outside the tumour that result in direct DNA/RNA damage through incorporation, undesired transport of activated compounds, variable expression of drug targets, and reduced levels of drug degradation.

Over a decade of publications exists regarding inherited genetic biomarkers of 5-FU-related toxicity, but only a handful of polymorphisms and rare genetic variants associated with toxicity have been identified with high confidence.^7^ These include two common polymorphisms in *TYMS* (5′VNTR 2R/3R and 3′UTR 6 bp ins-del) and rare functionally deleterious *DPYD* variants, chiefly *2A and 2846T>A.^7^ ^8^ In aggregate, these variants are potentially useful, but suboptimal, for the prediction of toxicity in clinical practice. Furthermore, there is only limited evidence that genetic variants are generalisable as predictors of toxicity across 5-FU regimens.^7^

In this study, we have investigated the 25 candidate genes that act in the capecitabine/5-FU pathway for new common and rare genetic variants that are associated with capecitabine toxicity. Our patient set comprises about 1000 individuals from the QUASAR2 trial of capecitabine±bevacizumab (Avastin, Genentech/Roche). Our results show that comprehensive genetic studies in sufficiently large, homogeneous datasets can identify additional toxicity predisposition variants in known candidate genes.

## Methods

### Patients

The QUASAR2 study (http://www.octo-oxford.org.uk/alltrials/infollowup/q2.html; http://www.controlled-trials.com/ISRCTN45133151/) is a phase III randomised controlled trial of adjuvant capecitabine (Xeloda) (1250 mg/m^2^ twice daily for 14 days every 3 weeks, total of 8 cycles) ± bevacizumab (Avastin) (7.5 mg/kg every 3 weeks) following resection of stage II/III CRC. Patients were entered into the study between July 2005 and December 2011 at 123 UK and 81 non-UK sites. Survival analyses are scheduled for 2014. Of 1119 patients with blood collected as of July 2010, 1046 were selected for genetic study based on availability of clinical data and informed consent. Patient characteristics are shown in table 1. All work was performed with full UK Ethics Committee approval, according to the tenets of the Declaration of Helsinki.

**Table 1:** QUASAR2 patient characteristics

	Number	%
Site		
Colon	930	89
Rectum	116	11
Stage		
II	365	35
III	681	65
Ethnicity		
Caucasian	1046	100
Sex		
Male	593	57
Female	453	43
Age, years		
Median	65	
Minimum	22	
Maximum	85	
WHO performance status		
0–1	1046	100
Treatment		
Capecitabine (cap)	496	47
Cap+bevacizumab	550	53
Grade 3+adverse events		
Global	353	34
Diarrhoea	109	10
HFS	247	24
Mucositis	11	1
Stomatitis	12	1
Vomiting	15	1
Neutropaenia	22	2
Thrombocytopaenia	4	0

### Toxicity phenotypes

Toxicity phenotype data were collected as part of QUASAR2 according to the NCI Common Toxicity Criteria for Adverse Events (CTCAE) V.3.0. Maximum toxicity (0–4) at any treatment cycle was derived for each of the following individual FU-related toxicities: diarrhoea, nausea and vomiting, mucositis/stomatitis, neutropaenia, thrombocytopaenia and HFS. We then derived a global measure of toxicity, defined as the maximum individual toxicity score measured for each patient. Global and individual toxicities were analysed using two approaches: (1) a binary classification into low toxicity (grade 0–2) versus high (dose-limiting) toxicity (grade 3–4) and (2) a quantitative measure of toxicity. In the latter, if <100 patients experienced a particular grade of toxicity, we combined them into a single bin with an adjacent grade. Specifically, we analysed grades (0–1 v 2 v 3–4) for global toxicity, diarrhoea and HFS and 0v1234 for the other, rarer toxicity phenotypes. Toxicity data by grade are shown in online supplementary table 2.

### Genotype data

Exclusion of patients on the basis of availability of DNA and completeness of toxicity data, non-Caucasian ethnicity or genotyping quality control (see online supplementary methods)^9^ meant that data were available from 940 patients genotyped using Illumina tagging SNP arrays and a largely overlapping set of 968 QUASAR2 patients genotyped on Illumina HumanExome12v1_A or -12v1-1_A arrays, which were designed to capture uncommon protein-coding variation.^10^ ^11^ For each of the 25 capecitabine/5-FU pathway genes (see online supplementary table 1), we identified genetic variants that were present on the arrays and that lay within 25 kb either side of the coding region of the longest isoform of each gene. We used imputation to obtain missing genotypes arising from differences in array content.^12–14^ Further genotyping was performed using previously described methods.^15–17^

For loci at which significant or borderline significant associations between genetic variants and toxicity were detected, we performed fine mapping studies by using the methods above to impute all SNPs in a 1.5 Mb flanking region, in order to refine the association signal.

### Statistical analysis

For each of the 1456 SNP and exome array variants, our primary analysis was to test associations between global (any 5-FU-related) dose-limiting (grade 012v34) toxicity and genotype. Frequentist tests under a missing data linear or logistic regression model were implemented using SNPTESTv2. Samples were stratified by QUASAR2 treatment with age and gender as covariates. Meta-analysis of the two arms of QUASAR2 was performed using GWAMA, including tests of interarm heterogeneity. A stringent Bonferroni-corrected p value threshold of 3.43×10^−5^ (=0.05/1456) was used to indicate a significant association for the primary analysis of (binary) global dose-limiting toxicity.

For selected SNPs with association signals that reached or approached formal significance, we imputed additional SNPs within 1.5 Mb flanking regions (see online supplementary methods). We performed association tests for global and specific toxicities using the global grade 012v34 measure. Since the genotyped and imputed SNPs were non-independent, we declared associations with imputed SNPs significant using the same threshold of p=3.43×10^−5^. For any region within which one or more SNPs achieved significant associations with global toxicity, the underlying individual toxicities were investigated at the most strongly associated SNPs using quantitative measures and clinically actionable cut-offs for dose delay or reduction in QUASAR2 (generally, grade 012v34, except for grade 01v234 for diarrhoea).

To test for independent effects of variants within a region, we used logistic regression analysis in R, with age, sex and study arm as covariates. The best-fitting model was determined as that which minimised the Akaike information criterion (AIC) subject to a variant showing an association at p=0.05. Haplotype analyses at *DPYD* and *TYMS/ENOSF1* were performed using the *--hap-logistic* and *--independent-effect* commands in PLINK. Tests to examine multiple genetic variants were performed in PLINK. Receiver operator characteristic (ROC) analysis was performed in Stata using a binary classification of patients to either grade 0/1/2 or grade 3/4 global toxicity, using a genetic score given for each individual by ΣβiNi, where βi is the beta coefficient of the ith SNP significantly associated with global toxicity in a logistic regression model, and Ni is the number of harmful alleles carried by that individual at that locus.

### Sequencing and validation of novel variants identified

Amplicon sequencing of the coding regions of *DPYD* and *TYMS* was performed by Roche/454 Titanium GS FLX technology in 100 patients with the highest levels of 5-FU-related toxicity (‘HiTox’), specifically grade 3 or grade 4 diarrhoea in the first 4 cycles of treatment and or other grade 3/4 toxicities in the first 4 cycles of treatment. We also sequenced the same amplicons in 100 patients with no adverse toxicity events during the entire duration of treatment (‘LoTox’). The missense *DPYD* variant p.Ala551Thr (A551 T) was identified in the HiTox pool. The DNA for each individual comprising the pool was Sanger-sequenced to identify those carrying this variant. Only one heterozygous individual was found. For analysis of the whole sample set, we designed KASPar (http://cshprotocols.cshlp.org/content/2007/9/pdb.prot4841.abstract) allele-specific single-nucleotide variant primers to detect A551 T and included three duplicates of the known variant sample in each run to facilitate genotype clustering (details available upon request). All samples that did not cluster with the A allele homozygotes were subsequently examined by bidirectional Sanger sequencing.

## Results

A total of 1046 capecitabine-treated patients from the QUASAR2 trial were initially selected. Grade 3+ global toxicity was observed in 34% of these patients, with severe diarrhoea in 10% and HFS in 24% (see online supplementary table 2). Severe toxicity was less common for the other phenotypes, although five patients experienced grade 4 neutropaenia. After exclusions (see online supplementary methods), genotype data were available for 940 patients genotyped on genome-wide tagSNPs arrays and 968 patients genotyped on the Exome array. Across the two types of arrays, we identified 1456 genetic variants that mapped to within 25 kb of the 25 capecitabine/5-FU candidate genes (see online supplementary table 1). In the primary analysis, we searched for associations between each genetic variant and global (grade 012v34) capecitabine toxicity. Rather than regarding the two arms of QUASAR2 as discovery and validation sets, with consequent reduction in statistical power, we performed a meta-analysis of data from the 439 patients in arm A (capecitabine) and the 501 patients in arm B (capecitabine+bevacizumab), and assessed evidence for between-arm heterogeneity. The results of this analysis on a per SNP and SNP set basis are summarised in supplementary tables S3–S5, and the most important findings for all genotyped or imputed SNPs showing a significant association (p<3.43×10^−5^) are described in detail below.

### New associations of common DPYD variants with capecitabine toxicity

**Figure 2:**
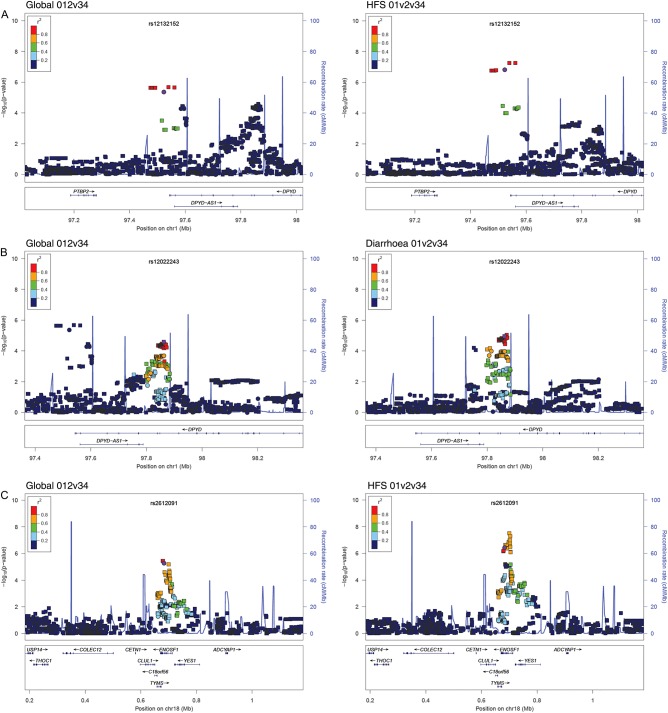
Regional plots of *DPYD* and *TYMS* SNPs for associations with capecitabine-related toxicity. Regional associations between polymorphisms and (left) global grade 012v34 capecitabine toxicity or (right) selected specific toxicities were plotted using LocusZoom and are shown for SNPs 500 kb either side of (a) rs12132152 which maps 3′ of *DPYD*, (b) rs12022243, mapping to an intron of *DPYD* and (c) rs2612091, which maps to an intron of *ENOSF1*. The x-axis shows chromosome position, while the y-axis shows the log_10_P for the association. Circles represent genotyped SNPs included in the 1456-SNP test panel, with the most significantly associated SNP in purple. Squares represent imputed SNPs. Linkage disequilibrium (calculated using hg19/1000 genomes, March 2012 EUR population) with the most significantly associated SNP is shown by colour as indicated.

We found that the A-allele (freq.=0.03) of SNP rs12132152 was associated with global capecitabine toxicity (OR_globalbinary_=3.83, p=4.31×10^−6^; table 2). rs12132152 is an intergenic SNP 22 kb downstream of *DPYD* (chr1:97523004, b37). Upon imputation of variants in the region flanking this tag SNP (see online supplementary methods), we identified a few SNPs with marginally more significant associations, notably rs76387818 (chr1:97 539 400; OR_binary_=4.05, p=2.11×10^−6^, r^2^_tag_=0.98; table 2; figure 2A). Results were similar when the quantitative measure of global toxicity was used (table 2). We investigated the individual phenotypes comprising the global toxicity measure. rs12132152 and rs76387818 were principally associated with HFS, under quantitative and binary models (for rs76387818, OR_hfsquant_=1.78, p=5.51×10^−8^, OR_hfsbinary_= 6.44, p=1.75×10^−8^), albeit with some weaker evidence of associations with diarrhoea (table 2).

**Table 2:** Selected associations between genetic variants and capecitabine toxicity in QUASAR2

Gene	SNP b37 coordinate	Toxicity- associated allele/other allele	TAF	n Genotyped n imputed	Info score ^A^=Hap370 ^B^=Hap610 ^C^=Omni2.5	Global binary: 012v34 OR (95% CI) p-value	Global quant:01v2v34 OR (95% CI) p-value	HFS binary:012v34 OR (95% CI) p-value	HFS quant:01v2v34 OR (95% CI) p-value	Diarrhoea binary:012v34 OR (95% CI) p-value	Diarrhoea quant:01v2v34 OR (95%CI) p-value	Other clinically actionable associations
*DPYD*	rs12132152 chr1:97523004	A/G	0.031	456 484	0.993^A^	**3.83 (3.26–4.40)**	**1.61 (1.41–1.82)**	**6.12 (5.48–6.76)**	**1.74 (1.53–1.95)**	0.44 (0–1.32)	0.85 (0.68–1.02)	
						**4.31×10^−6^**	**5.89×10^−6^**	**3.29×10^−8^**	**1.47×10^−7^**	0.065	0.068	
*DPYD*	rs76387818 chr1:97539400	A/G	0.031	0 940	0.993^A^ 0.999^B^ 0.999^C^	**4.05 (3.47–4.62)**	**1.66** **(1.45–1.87)**	**6.44** **(5.79–7.09)**	**1.78** **(1.57–1.99)**	0.44 (0–1.33)	0.86 (0.68–1.03)	
						**2.11×10^−6^**	**1.93×10^−6^**	**1.75×10^−8^**	**5.51×10^−8^**	0.071	0.083	
*DPYD*	rs7548189 chr1:97 867 713	A/C	0.196	940 0	N/A	1.67 (1.43–1.91)	**1.23 (1.14–1.31)**	1.42 (1.15–1.69)	1.16 (1.07–1.25)	1.21 (0.84–1.58)	**1.18 (1.10–1.25)**	**Diarrhoea 01v234** **1.76 (1.50–2.02)**
						3.79×10^−5^	**6.82×10^−6^**	0.011	0.0011	0.0015	**1.54×10^−5^**	**1.72×10^−5^**
*DPYD*	rs12 022 243 chr1: 97 862 780	T/C	0.196	0 940	0.996^A^ 0.992^B^ 0.998^C^	**1.69 (1.45–1.94)**	**1.23 (1.14–1.32)**	1.43 (1.16–1.7)	1.16 (1.07–1.25)	**1.79 (1.54–2.05)**	**1.18 (1.11–1.26)**	
						**2.55 x10^−5^**	**4.45 x10^−6^**	0.0096	8.26 x10^−4^	**9.86 x10^−6^**	**1.11 x10^−5^**	
*TYMS*/*ENOSF1*	rs2612091 chr18:683 607	C/T	0.532	940 0	N/A	**1.59 (1.39–1.79)** **5.28×10^−6^**	**1.19 (0.77–0.91)** **2.35×10^−6^**	**1.57 (0.45–0.83)** **2.94×10^−6^**	**1.21 (0.76–0.90)** **3.67×10^−7^**	1.18 (0.55–1.15) 0.29	1.04 (0.90–1.03) 0.27	**HFS 01v234** **1.57 (0.45–0.83)** **2.94×10^−6^**
*TYMS*/*ENOSF1*	rs2741171 chr18:700 687	T/C	0.534	0 940	0.960^A^ 0.975^B^ 0.990^C^	**1.60 (1.39–1.80)** **6.64×10^−6^**	**1.20 (1.13–1.28)** **9.24×10^−7^**	**1.74 (1.51–1.97)** **1.64×10^−6^**	**1.23 (1.16–1.31)** **3.10×10^−8^**	1.01 (0.70–1.32) 0.92	1.03 (0.97–1.09) 0.37	**HFS 01v234** **1.61 (1.42–1.80)** **1.44×10^−6^**

The table shows the results of the meta-analysis of global and selected individual toxicities, measured as binary or continuous (‘quant’) variables, in the two arms of QUASAR2. The frequency of the toxicity-associated allele (TAF) is also shown and ORs are expressed relative to this. Imputation quality Info Score is also shown, as are numbers of samples imputed and directly genotyped. The final column shows results for toxicity phenotype classifications that could lead to treatment change or delay according to the QUASAR2 protocol. There was no evidence of heterogeneity between QUASAR2 arms in any of these analyses P_het_>0.2, I^2^<0.25).

We then investigated in silico possible functional mechanisms underlying the rs12132152/rs76387818 association. We examined ENCODE data (http://genome.ucsc.edu/ENCODE/) for the region containing rs12132152 and seven strongly correlated SNPs (figure 2A: approximately chr1:97 475 000-97 562 000, b37). FAIRE and histone K4 methylation data suggested that this is a region of open chromatin and one correlated SNP in particular, rs12123160, lies at a methylated CpG. Although no suitable data from normal liver were available, we found that genotypes at rs12132152 and correlated SNPs were not associated with *DPYD* expression in adipose tissue, lymphoblastoid cells or skin (Genevar database, p>0.13)^18^ or in colon tissue (TCGA, p=0.72).

The second *DPYD* toxicity-associated variant (table 2) was identified following SNP imputation in the region of 1.5 Mb surrounding rs7548189, a tagSNP intronic to *DPYD* (chr1:97 867 713, b37). rs7548189 was borderline associated with the binary meaure global toxicity (OR_globalbinary_=1.67, p=3.79×10^−5^). Further investigation showed rs7548189 formally to be associated with the quantitative measure of global toxicity, and with diarrhoea under binary and quantitative models (OR_globalquant_=1.23, p=6.82×10^−6^; OR_diarrhoeaquant_=1.18, p=1.54×10^−5^; OR_diarrhoeabinary_=1.76, p=1.72×10^−5^). As can be seen from table 2, HFS also contributed to the association with global toxicity. Following regional imputation, rs12022243 (r^2^ with rs7548189=0.95) was found formally to be associated with global toxicity under a binary model (OR_globalbinary_=1.69, p=2.55×10^−5^). We found that rs12022243 showed excellent imputation quality: of 190 independently assessed individuals, only 4 (2%) genotypes were missing, and all the remaining genotypes were imputed correctly (see online supplementary methods).

We found little evidence from ENCODE data that rs7548189 is functional, but rs12022243 falls in a region of open chromatin that may have enhancer activity. rs7548189 was not associated with *DPYD* expression levels in lymphoblasts, fibroblasts, T-cells, adipose tissue, or skin on the Genevar database (p>0.07)^18–20^ or in colon tissue from TCGA (p=0.97). rs12022243 Was absent from these datasets.

Using logistic regression analysis, we found that the rs12132152 and rs7548189/rs120222243 signals were independent of each other and of the known *DPYD* toxicity variants *2A (rs3918290) and 2846T>A (rs67373796) (details not shown; linkage disequilibrium data in online supplementary figure 1). Further analysis of haplotypes based on 81 tagSNPs in a 25 kb window either side of *DPYD* provided no further refinement of the rs12132152 and rs7548189/rs120222243 associations and showed no evidence of additional, independent association signals (details not shown).

### Resolving the toxicity associations at the TYMS and ENSOF1 loci

We found that the G-allele (freq.=0.45) of SNP rs2612091 was associated with increased global toxicity (OR_globalbinary_=1.59, p=5.28×10^−6^; OR_globalquant_=1.19, p=2.35×10^−6^; table 2). rs2612091 lies 10 kb downstream of *TYMS* within an intron of enolase superfamily member 1 (*ENOSF1*, chr18:683 607). Fine mapping showed somewhat more significant associations for a SNP, rs2741171 (chr18:700 687), in linkage disequilibrium (r^2^=0.73) with rs2612091, particularly for the quantitative measure of global toxicity and HFS (OR_global_ _quant_=1.2, p=9.24×10^−7^). The rs2741171 variant is further downstream of *TYMS* (27 kb) and again is intronic for *ENOSF1*, but both SNPs fall between recombination hotspots that flank the entirety of *TYMS* and *ENOSF1* (figure 2B). The rs2612091/rs2741171 effect on toxicity was essentially driven by HFS, with an especially strong association being observed using the quantitative measure (rs2612091: OR_hfsquant_=1.21, p=3.67×10^−7^; rs2741171: OR_hfsquant_=1.23, p=3.10×10^−8^).

*ENOSF1* is a largely uncharacterised gene that appears to encode a protein and RNAs antisense to *TYMS*. It has been proposed that *ENOSF1* regulates *TYMS* mRNA and/or protein expression.^21^ We therefore analysed associations between rs2612091 genotype and *TYMS* and *ENSOF1* expression using the Genevar and TCGA databases. Using Caucasian-matched twin data,^18^ the rs2612091 G-allele significantly decreased *ENOSF1* expression in adipose tissue for both twin sets (p_set1_=7.0×10^−4^, p_tset2_=9.7×10^−6^) and in lymphoblastoid cells for one set (p_set1_=0.89, p_set2_=0.0012). However, rs2612091 genotype was not associated with *TYMS* expression (p>0.30 for each of the same analyses). Similarly, in lymphoblastoid cell line expression data,^20^ the rs2612091 G-allele was associated with decreased expression of *ENOSF1* (p=1.9×10^−6^) but not *TYMS* (p=0.82). These results were further replicated in the TCGA colon data, in which the rs2612091 G-allele was again associated with decreased *ENOSF1* expression (OR=0.76, p=1.5×10^−7^), but not with *TYMS* (OR=0.95, p=0.45).

We tested the relationship of rs2612091 to two *TYMS* polymorphisms (5′ VNTR 2R/3R and 3′UTR 6 bp ins-del^22^ ^23^ that have previously been reported to alter *TYMS* expression, and hence, to affect 5-FU-related toxicity (see online supplementary table S4). Noting moderate LD between rs2612091 and the 5′VNTR and 3′UTR polymorphisms (r^2^=0.40 and 0.32; online supplementary figure 1), we tested whether the rs2612091 signal was independent of the 5′VNTR and 3′UTR polymorphisms, using the quantitative measure of HFS (01v2v34) as the toxicity phenotype, because HFS underlies the global toxicity signal observed for all three variants.^7^ First, the three variants were incorporated into a covariate-adjusted logistic regression model. Only rs2612091 remained significantly associated when adjusting for the other variants (for rs2612091 OR_hfsquant_=1.21, p=0.00049, 5′ VNTR p=0.19, 3′UTR p=0.48). Using rs2612091 alone in the model minimised the AIC. Second, we tested for evidence of interaction (epistasis) among the variants, but found none between rs2612091 and either the 5′VNTR (p=0.92) or the 3′UTR (p=0.19). These analyses suggested that rs2612091 and the previously identified variants do not act as a 3-polymorphism tag for unidentified variants, and that rs2612091 alone captures the association signal created by all three variants. Finally, we tested the independence of each individual polymorphism in a 3-polymorphism haplotype (see online supplementary table S4). We found that the G-allele of rs2612091 consistently increased the risk of toxicity, irrespective of the 5′VNTR or 3′UTR genotype (p=0.0021). Conversely, neither the 5′VNTR (p=0.17) nor the 3′UTR (p=0.61) risk-allele consistently increased risk of toxicity when varying the genotype of the other two SNPs. The analysis was repeated as 2-polymorphism haplotypes comprising rs2612091 and either the 5′VNTR or 3′UTR variant. Irrespective of either genotype at either the 5′VNTR or 3′UTR polymorphism, rs2612091 genotype was again significantly associated with HFS, (p=0.00053 and p=1.47×10^−6^ when incorporating 5′VNTRand 3′UTR, respectively). The above analyses were repeated using global toxicity, and all the above results were similar but reduced modestly in significance (data not shown).

Equivalent logistic regression and haplotype analysis using the top fine-mapping SNP rs2741171 showed even stronger evidence that the new SNP signal alone explained the associations at *TYMS/ENOSF1* (data not shown). We further tested the various combinations of rs2741171, rs2612091, 5′VNTR and 3′UTR polymorphisms in a multivariate logistic regression model and found that the model which minimised AIC incorporated rs2741171 alone (details not shown). rs2741171 lies next to a region of open chromatin that may be a p300 binding site.

### Identifying and assessing rare susceptibility variants in DPYD and TYMS

We sequenced the coding regions of *DPYD* and *TYMS* in pools of Hi-Tox and Lo-Tox patients (see online supplementary figures S2 and S3). In the HiTox pool, we identified a single missense variant that was not present on SNP and exome arrays (*DPYD* c.G1651A; p.Ala551Thr; chr1:97 981 371). We found no other occurrence of this variant in the full set of 968 patients. A551 T was predicted to be strongly damaging by SIFT, Polyphen, PhyloP and MutationTaster, and the single patient with this allele had experienced grade 4 neutropaenia and thrombocytopaenia. Database searches determined that this variant has been previously reported as causal for DPYD Deficiency Syndrome (OMIM 612779).^24^ We confirmed that A551 T was not in linkage disequilibrium with any of the other common or rare *DPYD* toxicity variants.

We then considered only the 19 patients with extreme (grade 4) toxicity at any cycle and determined which alleles they carried at the three toxicity SNPs and their complement of rare *DPYD* alleles from the literature, including 2846 A>T and *2A that were shown to be associated with 5-FU toxicity in our previous meta-analysis^7^ (table 3). There was no good evidence that the risk alleles at the three new toxicity SNPs were over-represented as a group in these 19 patients (table 3). Using the evaluation of Caudle *et al*^8^ as a guide, supplemented by data from this study, we then assessed the likely contributions of each rare *DPYD* variant to extreme toxicity. There was insufficient prior evidence^8^ to regard six *DPYD* alleles (*4, *5, *6, *9A, M166 V and K259E) as pathogenic (see online supplementary table S6). Inspection of the genotypes of these polymorphisms in the severe toxicity cases (table 3) and the tests of association with binary global toxicity (see online supplementary tables S3 and S6) did not contradict this view. Several other rare *DPYD* alleles (*3, *7, *8, *9B, *10, *11, *12) were not present in our sample set. We denoted four rare *DPYD* alleles as severely functionally deleterious: *2A, 2846T>A, *13 and A551 T. Five of 19 (26%) severe-toxicity patients carried one of these *DPYD* alleles (table 3). Of these five cases, four (80%) had life-threatening bone marrow toxicity (G4 neutropaenia and thrombocytopaenia), whereas the other had G4 diarrhoea. Another individual with G4 neutropaenia, but not thrombocytopaenia, did not carry any of the four *DPYD* alleles. Overall, for prediction of severe myelosuppression, the rare *DPYD* variants had 83% sensitivity, 99% specificity, 29% positive predictive value and 99.9% negative predictive value.

**Table 3:** Genotypes of QUASAR2 individuals with grade 4 toxicity at selected DPYD variants

Case	D	V	H	N	P	M	S	Possible explanation	2846	*2A	A551T	*13	*4	*5	*6	*9A	M166V	K259E	rs12132152	rs12022243	rs2612091
1	**4**	0	2	0	0	0	0		0	0	0	0	0		0	0	0	0	0	0	2
2	**4**	0	0	0	0	0	0		0	0	0	0	0	0	0	0	0	0	0	1	2
3	0	**4**	0	0	0	0	0		0	0	0	0	1	1	0	0	0	0	0	1	1
4	**4**	2	2	0	0	0	0		0	0	0	0	0	0	0	2	1	0	0	0	0
5	**4**	0	0	0	0	0	1	2846T>A	1	0	0	0	1	1	0	0	0	0	0	0	0
6	2		1	**4**	**4**	0	2	2846T>A	1	0	0	0	1	0	0	0	0	0	0	2	1
7	3	0	2	**4**	**4**	3	3	A551T	0	0	1	0	0	0	0	0	0	0	0	1	2
8	3	1	3	**4**	2	3	3		0	0	0	0	0	2	0	0	0	0	0	1	2
9	**4**	0	2	0	0	1	0		0	0	0	0	1	0	0	0	0	0	0	1	2
10	**4**	1	3	0	0	1	0		0	0	0	0	0	0	0	0	0	0	0	1	2
11	**4**	2	1	1	0	0	0		0	0	0		0	1	0	0	0		0	1	2
12	0	0	3	**4**	**4**	3	3	*2A	0	1	0	0	0	0	0	1	1	0	0	0	1
13	**4**	0	1	0	1	1	1		0	0	0	0	0	1	0	0	0	0	0	1	1
14	0	**4**	3	0	1	0	0		0	0	0	0	0	0	0	1	1	0	0	1	2
15	**4**	0	1	0	0	1	1		0	0	0	0	0	1	0	0	0	0	0	0	1
16	**4**	0	2	0	0	0	0		0	0	0	0	0	0	0	0	0	0	0	1	1
17	1	0	**4**	0	0	0	0		0		0	0	0	0	0	0	0	0	0	1	0
18	2	**4**	3	0	0	0	1		0	0	0	0	0	1	0	0	0	0	0	0	0
19	3	0	2	**4**	**4**	0	**4**	*13	0	0	0	1	0		0			0			

The variants shown are (1) those identified by this study (rs12132152, rs12022243, rs2612091, *DPYD* A551 T), (2) *DPYD* alleles (2846 A>T and *2A) shown to be associated with 5-FU toxicity in the meta-analysis of Rosmarin *et al*^7^ and (3) potential *DPYD* toxicity alleles from Caudle *et al*.^8^ Genotypes shown are major allele homozygote (0), heterozygote (1) and minor or variant allele homozygote (2). Blank cells denote missing data. The allele that provides a plausible explanation for the severe toxicity is shown. Note that *4 and *5 *DPYD* alleles are in complete linkage disequilibrum (D’=1.0) with 2A or 2846T>A. Overall association statistics and genotype frequencies in cases and controls are shown for the analysis of binary global toxicity in online supplementary table S3.

For the toxicities, D=diarrhoea; H=HFS; M=mucositis; N=neutropaenia; P=thrombocytpaenia; S=stomatitis; V=vomiting.

## Discussion

Through the analysis of capecitabine-treated patients from the QUASAR2 trial for SNPs and rare genetic variants in 25 capecitabine/5-FU pathway genes and subsequent exon sequencing of *DPYD* and *TYMS*, we have identified new genetic predictors of capecitabine-induced toxicity and clarified the origins of previously reported signals. Two of the new toxicity variants (rs12022243 and rs12132152) map to *DPYD* and are independent of previously reported *DPYD* toxicity alleles*.* The toxicity-associated allele at rs12022243 is common (freq.=0.22) and has a moderate effect (OR_globalbinary_=1.8) on toxicity risk, influencing HFS and diarrhoea. The rs12132152 toxicity allele is less common, but not rare (freq.=0.03), and its effect is greater (OR_globalbinary_=3.8). Although associations between other common *DPYD* polymorphisms and 5-FU toxicity have previously been proposed,^8^ ^23^ we failed to confirm these in this analysis and in a previous meta-analysis of multiple datasets.^7^

Through sequencing patients with high capecitabine toxicity, we identified a third new *DPYD* toxicity allele, p.Ala551Thr.^24^ Like the other rare *DPYD* variants (*2A and 2846T>A) that have established associations with 5-FU-related toxicity in the heterozygous state, A551 T has been shown to cause the recessive DPYD deficiency syndrome when present as the homozygote or compound heterozygote with another mutant allele. The identification of A551 T in a patient experiencing grade 4 neutropaenia and thrombocytopaenia adds further support to the view that all rare *DPYD* variants that cause DPYD Deficiency Syndrome greatly increase the risk of 5-FU toxicity in heterozygotes. Furthermore, of the three other QUASAR2 patients who experienced grade 4 myelotoxicity—two of whom were the only toxicity-induced deaths in QUASAR2—one carried *DPYD* *2A, one 2846A>C and one *13. Of the 12 carriers of rare, functionally deleterious *DPYD* alleles who did not develop severe toxicity, seven suffered a grade 3 toxicity and may therefore have been spared severe toxicity by capecitabine dose reduction. However, the reason for the remaining five individuals avoiding clinically important toxicity is unclear. Much interpatient difference in 5-FU toxicity remains unexplained, and it remains very plausible that some of this variability is heritable.

Our finding of another capecitabine toxicity SNP, rs2612091, produced some very unexpected results. This common variant is downstream of *TYMS* and intronic to *ENOSF1*. Our detailed analysis showed that the rs2612091 signal appears to explain both the previously reported associations between 5-FU/capecitabine toxicity and *TYMS* polymorphisms (5′VNTR 2R/3R and 3′UTR 6 bp ins-del)*.* This is especially surprising given published evidence that the two *TYMS* polymorphisms directly affect *TYMS* mRNA expression and protein levels. However, recent critical assessments have shown that the data linking *TYMS* expression to 5′VNTR 2R/3R and 3′UTR 6 bp ins-del genotypes are actually very mixed.^25^ In fact, our analysis of public mRNA expression data demonstrated rs2612091 to be associated with *ENOSF1* expression and not with *TYMS* expression. Our data imply that the *TYMS* 5′VNTR and 3′UTR toxicity association signals result from LD between these polymorphisms and tagSNP rs2612091 or, more likely, fine-map SNP rs2741171 (see online supplementary figure 1A). We therefore conclude that *ENOSF1* is most likely to be the target of the functional variation tagged by rs2612091. *ENOSF1* and *TYMS* transcripts are overlapping, but toxicity SNP does not appear to act through antisense-mediated down-regulation of *TYMS* mRNA. However, ENOSF1 protein has been proposed as an influence on TYMS activity, and this remains a plausible mechanism of toxicity.

We found no evidence of heterogeneity between the two arms of QUASAR2 in terms of the effects of genetic variants on global toxicity or any specific toxicity. The toxicity profiles of capecitabine and bevacizumab principally overlap through the risk of HFS, although the effects of the former greatly outweigh those of the latter (estimated 18% vs 7% for QUASAR2). In principle, a polymorphism could predispose to HFS caused by either capecitabine and/or bevacizumab. However, such a variant is most likely to act at the level of the HFS target tissue, whereas, our variants were specifically chosen for potential effects on 5-FU metabolism. The most likely effect of the use of bevacizumab on our study was, therefore, a small decrease in statistical power owing to a higher ‘background’ level of HFS resulting from a non-capecitabine source in arm B.

In summary, we have identified four new variants associated with capecitabine toxicity. Further work is desirable in order to confirm and quantitate these associations in additional datasets, and to understand the mechanistic origins of capecitabine-related toxicity, especially for the *TYMS* and *ENOSF1* loci. While not yet an ideal test for clinical use (see online supplementary figure 4), genetic testing can be used to highlight patients at increased risk of capecitabine toxicity. A two-tier test may be justifiable, comprising (1) a sensitive test for severe, life-threatening toxicity based principally on rare *DPYD* variants and (2) a test additionally incorporating SNPs to highlight the risk of clinically actionable toxicity. It is a moot point as to whether such a strategy would currently be cost effective, and it is not yet known how well our panel of variants predicts toxicity from 5-FU delivered by other means or in combination regimens. Although our dataset was relatively large, and power was good to detect toxicity variants with relatively large effects (typically >80% power for a common variant conferring an OR>1.5), power was very limited to detect variants with lower allele frequency and/or smaller effect size (see online supplementary table S3). Further efforts to identify additional polymorphisms and rare variants associated with 5-FU toxicity remain valid.

## URLs

Primer3: http://sourceforge.net/projects/primer3/

LocusZoom: http://csg.sph.umich.edu/locuszoom/

SNPTEST2: https://mathgen.stats.ox.ac.uk/genetics_software/snptest/snptest.html

IMPUTE2: http://mathgen.stats.ox.ac.uk/impute/impute_v2.html

GWAMA: http://www.well.ox.ac.uk/gwama/download.shtml

TCGA: http://tcga-data.nci.nih.gov/

## Supplementary Material

Web supplement
