# Supplementary material for: A candidate gene study of capecitabine-related toxicity in colorectal cancer identifies new toxicity variants at DPYD and a putative role for ENOSF1 rather than TYMS
Source: Gut. 2014 Mar 19;64(1):111–20. doi: 10.1136/gutjnl-2013-306571 (PMC4283622; doi:10.1136/gutjnl-2013-306571)
Supplement: Web supplement [file gutjnl-2013-306571-s1.pdf]

Supplementary Table 1. Candidate gene region summary

Gene Symbol	Gene Name	Location – Build 37 (coordinates do not include 25kb flanking region)	Number of Test Panel SNPs
ABCB1	ATP-binding cassette, sub-family B	chr7:87132948-87342564	77
ABCC3	ATP-binding cassette, sub-family C, member 3	chr17:48712218-48769063	64
ABCC4	ATP-binding cassette, sub-family C, member 4	chr13:95672083-95953687	224
ABCC5	ATP-binding cassette, sub-family C, member 5	chr3:183637724-183735727	101
ABCG2	ATP-binding cassette, sub-family G, member 2	chr4:89011416-89152474	57
CDA	cytidine deaminase	chr1:20915444-20945400	26
CES1	carboxylesterase 1 isoform a precursor	chr16:55836764-55867075	24
CES2	carboxylesterase 2 isoform a precursor	chr16:66968347-66978994	59
DPYD	dihydropyrimidine dehydrogenase	chr1:97543300-98386615	239
DPYS	dihydropyrimidinase	chr8:105391652-105479277	69
MTHFR	methylenetetrahydrofolate reductase	chr1:11845787-11866115	38
PPAT	phosphoribosyl pyrophosphate amidotransferase	chr4:57259529-57301845	29
RRM1	ribonucleoside-diphosphate reductase subunit 1	chr11:4115924-4160106	29
RRM2	ribonucleoside-diphosphate reductase subunit 2	chr2:10262735-10270623	19
SLC22A7	solute carrier family 22 member 7 isoform b	chr6:43265998-43273276	26
SLC29A1	equilibrative nucleoside transporter 1	chr6:44187242-44201888	26
TK1	thymidine kinase 1	chr17:76170160-76183285	35
TYMP	thymidine phosphorylase	chr22:50964182-50968258	92
TYMS	thymidylate synthetase	chr18:657604-673499	34
UCK1	uridine-cytidine kinase 1 isoform a	chr9:134399191-134406655	43
UCK2	uridine-cytidine kinase 2 isoform a	chr1:165796890-165877339	22
UMPS	uridine monophosphate synthase	chr3:124449213-124464040	34
UPB1	beta-ureidopropionase	chr22:24890077-24922553	30
UPP1	uridine phosphorylase 1	chr7:48128355-48148330	16
UPP2	uridine phosphorylase 2	chr2:158851691-158992478	43

Supplementary Table 2. Toxicity frequencies in QUASAR2

Adverse Event	CTCAE grade	Patients
Global	0	75
	1	241
	2	375
	3	334
	4	19
	Unreported	2
Diarrhoea	0	370
	1	388
	2	175
	3	99
	4	10
	Unreported	4
Handfoot	0	176
	1	287
	2	331
	3	246
	4	1
	Unreported	5
Mucositis	0	734
	1	248
	2	49
	3	11
	4	0
	Unreported	4
Stomatitis	0	718
	1	244
	2	67
	3	11
	4	1
	Unreported	5
Vomiting	0	817
	1	134
	2	74
	3	12
	4	3
	Unreported	6
Neutropaenia	0	921
	1	71
	2	28
	3	17
	4	5
	Unreported	4
Thrombocytopaenia	0	961
	1	67
	2	9
	3	0
	4	4
	Unreported	5

Supplementary Table 3. Summary of results of association test between 1,456 5-FU pathway genetic variants and binary global toxicity. Note that the unproven possibility of additional, unreported independent risk SNPs at *DPYD* remains (e.g. rs10875047 showed an association at a significance level close to the P<3.43 x 10^{-5} threshold used).There was very little evidence of between-arm heterogeneity in SNP effects as shown by the I^2 statistic which is <75% in all cases.

rs number	chromosome	position	Risk allele	Alternative allele	OR (95% CI)	P value	I^2 (heterogeneity)	r^2 with one of the lead SNPs (lead SNP #)
**310/370CNV/610/Omni2.5 arrays**								
rs12132152	1	97523004	A	G	3.83 (2.16-6.79)	4.31X 10^{-6}	0.16	Lead SNP 1
rs2612091	18	683607	C	T	1.59 (1.30-1.92)	5.28X 10^{-6}	0	Lead SNP 2
rs7548189	1	97867713	A	C	1.67 (1.31-2.13)	3.79X 10^{-5}	0	Lead SNP 3
rs4495747	1	97855607	G	A	1.67 (1.31-2.13)	3.91X 10^{-5}	0	1.0 (3)
rs12021567	1	97856946	T	C	1.67 (1.31-2.13)	4.09X 10^{-5}	0	1.0 (3)
rs12040763	1	97857061	C	T	1.67 (1.31-2.13)	4.09X 10^{-5}	0	1.0 (3)
rs12043125	1	97857145	T	G	1.67 (1.31-2.13)	4.09X 10^{-5}	0	0.9 (3)
rs1112314	1	97850697	T	C	1.67 (1.31-2.13)	4.20X 10^{-5}	0	1.0 (3)
rs1356917	1	97852258	C	A	1.66 (1.30-2.12)	5.05X 10^{-5}	0	1.0 (3)
**rs10875047**	**1**	**97594994**	**C**	**T**	**1.70 (1.31-2.19)**	**5.28X 10^{-5}**	**0**	**0.1 (1)**
rs11165784	1	97584685	G	C	1.70 (1.31-2.19)	5.30X 10^{-5}	0	0.2 (1)
rs12566907	1	97862237	C	T	1.54 (1.23-1.93)	2.05X 10^{-4}	0	0.8 (3)
rs7540201	1	97860321	A	C	1.54 (1.22-1.93)	2.17X 10^{-4}	0	0.8 (3)
rs11799399	1	97604531	T	C	1.63 (1.26-2.11)	2.21X 10^{-4}	0	0.1 (1)
rs10875076	1	97837564	T	C	1.52 (1.21-1.91)	3.07X 10^{-4}	0	0.8 (3)
rs1415683	1	97845053	G	T	1.52 (1.21-1.91)	3.12X 10^{-4}	0	0.8 (3)
rs1890138	1	97839016	G	A	1.52 (1.21-1.91)	3.42X 10^{-4}	0	0.8 (3)
rs12039249	1	97603679	C	T	1.58 (1.23-2.03)	3.48X 10^{-4}	0	0.1 (1)
rs1709409	1	97602726	C	T	1.55 (1.21-1.99)	4.81X 10^{-4}	0	0.1 (1)
rs1760217	1	97602994	G	A	1.54 (1.20-1.97)	6.06X 10^{-4}	0	0.1 (1)

rs number	chromosome	position	Risk allele	Alternative allele	OR (95% CI)	P value	I^2 (heterogeneity)	r^2 with one of the lead SNPs (lead SNP #)
rs11165779	1	97564650	T	C	2.00 (1.32-3.01)	1.03X 10^{-3}	0	0.8 (1)
rs4434871	1	97873007	C	T	1.57 (1.20-2.07)	1.24X 10^{-3}	0	0.5 (3)
rs2244500	18	661005	A	G	1.37 (1.67-1.14)	1.26X 10^{-3}	0	0.6 (2)
rs12563828	1	97830721	T	A	1.41 (1.13-1.77)	2.63X 10^{-3}	0	0.4 (3)
rs10875071	1	97814678	C	T	1.47 (1.14-1.89)	2.70X 10^{-3}	0	0.6 (3)
rs11165845	1	97819405	A	G	1.39 (1.11-1.75)	3.41X 10^{-3}	0	0.4 (3)
rs2606246	18	678847	T	C	1.43 (1.79-1.12)	3.57X 10^{-3}	0	0.3 (2)
rs6678858	1	97878565	T	A	1.44 (1.11-1.86)	6.08X 10^{-3}	0	0.2 (3)
rs1879375	1	97807335	G	A	1.41 (1.10-1.81)	6.62X 10^{-3}	0	0.6 (3)
rs4497250	1	97882933	A	G	1.40 (1.10-1.78)	6.67X 10^{-3}	0	0.3 (3)
rs2847154	18	687270	G	A	1.37 (1.09-1.75)	7.02X 10^{-3}	0	0.3 (2)
rs7556439	1	97771947	A	C	1.35 (1.08-1.68)	8.25X 10^{-3}	0	0.2 (3)
rs628959	1	97738860	C	T	1.35 (1.08-1.69)	8.45X 10^{-3}	0	0.2 (3)
rs507170	1	97738354	G	C	1.35 (1.08-1.69)	8.47X 10^{-3}	0	0.2 (3)
rs2612081	18	695030	G	A	1.37 (1.09-1.72)	8.48X 10^{-3}	0	0.3 (2)
rs644428	1	97737704	C	T	1.35 (1.08-1.69)	8.51X 10^{-3}	0	0.2 (3)
rs553388	1	97737348	C	T	1.35 (1.08-1.69)	8.51X 10^{-3}	0	0.2 (3)
rs526645	1	97749380	G	A	1.33 (1.08-1.67)	9.18X 10^{-3}	0	0.2 (3)
rs1609519	1	97781039	G	A	1.34 (1.07-1.67)	1.01X 10^{-2}	0	0.2 (3)
rs13233308	7	87244960	T	C	1.30 (1.06-1.58)	1.07X 10^{-2}	0	Lead SNP 4
rs10875061	1	97744048	G	A	1.28 (1.05-1.56)	1.56X 10^{-2}	0	0.1 (3)
rs11165837	1	97759020	T	A	1.37 (1.09-1.75)	1.58X 10^{-2}	0	0.1 (3)
rs11165827	1	97739908	A	T	1.28 (1.04-1.56)	1.65X 10^{-2}	0	0.1 (3)
rs10783058	1	97761972	T	C	1.28 (1.04-1.56)	1.75X 10^{-2}	0	0.1 (3)
rs10783057	1	97730626	G	A	1.28 (1.04-1.56)	1.78X 10^{-2}	0	0.1 (3)
rs9782950	1	97803724	C	T	1.30 (1.05-1.62)	1.78X 10^{-2}	0	0.3 (3)

rs number	chromosome	position	Risk allele	Alternative allele	OR (95% CI)	P value	I^2 (heterogeneity)	r^2 with one of the lead SNPs (lead SNP #)
rs7522938	1	97727820	G	C	1.28 (1.04-1.56)	$1.85 X 10^{-2}$	0	0.1 (3)
rs12031561	1	97903583	G	A	1.26 (1.04-1.53)	$1.99 X 10^{-2}$	0.38	0.1 (3)
rs641805	1	97743805	A	T	1.27 (1.03-1.54)	$2.15 X 10^{-2}$	0	0.1 (3)
rs12726453	1	97750189	C	T	1.26 (1.03-1.54)	$2.17 X 10^{-2}$	0	0.1 (3)
rs11165875	1	97915213	C	T	1.25 (1.03-1.52)	$2.28 X 10^{-2}$	0	0.1 (3)
rs6593642	1	97750838	T	C	1.26 (1.03-1.53)	$2.36 X 10^{-2}$	0	0.1 (3)
rs4148424	13	95931490	G	A	1.31 (1.04-1.67)	$2.36 X 10^{-2}$	0	Lead SNP 5
rs614664	3	124486993	A	C	1.26 (1.03-1.54)	$2.41 X 10^{-2}$	0.64	Lead SNP 6
rs1729788	13	95808003	G	A	1.27 (1.03-1.58)	$2.62 X 10^{-2}$	0	0.04 (5)
rs2725256	4	89050998	G	A	1.25 (1.03-1.52)	$2.70 X 10^{-2}$	0	Lead SNP 7
rs13336470	16	66999370	G	A	1.36 (1.04-1.79)	$2.73 X 10^{-2}$	0	Lead SNP 8
rs12028565	1	97894619	C	T	1.24 (1.02-1.51)	$2.87 X 10^{-2}$	0	0.1 (3)
rs1564481	4	89061265	T	C	1.24 (1.02-1.52)	$2.88 X 10^{-2}$	0	1 (7)
rs11873007	18	680380	C	T	1.27 (1.02-1.56)	$2.95 X 10^{-2}$	0	0.3 (2)
rs3819101	18	677240	G	A	1.27 (1.02-1.56)	$2.96 X 10^{-2}$	0	0.3 (2)
rs12535512	7	87220334	C	T	1.25 (1.02-1.52)	$3.01 X 10^{-2}$	0	0.8 (4)
rs3786355	18	681962	G	A	1.27 (1.02-1.56)	$3.13 X 10^{-2}$	0	0.3 (2)
rs7325861	13	95912228	T	G	2.04 (1.06-3.85)	$3.25 X 10^{-2}$	0	0.04 (5)
rs528455	1	97749198	T	C	1.23 (1.02-1.52)	$3.30 X 10^{-2}$	0	0.1 (3)
rs4148733	7	87213232	A	G	1.37 (1.03-1.85)	$3.32 X 10^{-2}$	0.45	0.1 (4)
rs12047910	1	97600039	A	G	1.35 (1.02-1.79)	$3.47 X 10^{-2}$	0	0.01 (1)
rs4693930	4	89122833	A	G	1.23 (1.01-1.50)	$3.53 X 10^{-2}$	0	0.1 (7)
rs4949952	1	97886171	T	C	1.23 (1.01-1.52)	$3.55 X 10^{-2}$	0.30	0.03 (1)
rs4148732	7	87234049	T	C	1.37 (1.02-1.85)	$3.81 X 10^{-2}$	0.43	0.1 (4)
rs2622629	4	89094064	C	T	1.23 (1.01-1.51)	$4.02 X 10^{-2}$	0	0.7 (7)
rs2766482	13	95785721	T	G	1.23 (1.01-1.51)	$4.09 X 10^{-2}$	0	0.04 (5)

rs number	chromosome	position	Risk allele	Alternative allele	OR (95% CI)	P value	I^2 (heterogeneity)	r^2 with one of the lead SNPs (lead SNP #)
rs3821536	3	124483952	C	T	1.30 (1.01-1.67)	4.20X 10^{-2}	0	0.4 (6)
rs2291081	3	124485235	G	A	1.30 (1.01-1.67)	4.26X 10^{-2}	0	0.4 (6)
rs899498	13	95804316	A	C	1.25 (1.01-1.54)	4.36X 10^{-2}	0	0.02 (5)
rs4148432	13	95913082	C	T	1.89 (1.02-3.45)	4.39X 10^{-2}	0	0.04 (5)
rs7986087	13	95915745	C	T	1.85 (1.02-3.33)	4.42X 10^{-2}	0	0.04 (5)
rs2853151	8	105396792	T	C	1.85 (1.01-3.33)	4.56X 10^{-2}	0	Lead SNP 9
rs7550959	1	97926839	G	A	1.22 (1.00-1.47)	4.59X 10^{-2}	0	0.1 (3)
rs2235035	7	87179086	G	A	1.23 (1.00-1.54)	4.68X 10^{-2}	0.17	0.2 (4)
rs1922240	7	87183354	T	C	1.23 (1.00-1.54)	4.72X 10^{-2}	0.17	0.2 (4)
rs1479390	13	95803139	T	G	1.24 (1.00-1.54)	4.84X 10^{-2}	0	0.06 (5)
rs2651204	6	43259087	T	C	1.41 (1.00-1.96)	4.85X 10^{-2}	0	Lead SNP 10
**Exome array**								
rs67376798	1	97547947	A	T	10.0 (2.50-33.3)	9.74X 10^{-4}	0	0 (1 and 3)
rs11165846	1	97819667	G	C	1.42 (1.14-1.78)	1.89X 10^{-3}	0	0.4 (3)
rs147266709	9	134398452	T	C	2.79 (1.42-5.47)	2.82X 10^{-3}	0.68	lead SNP 11
rs9616787	22	50943506	T	C	2.90 (1.34-6.28)	6.89X 10^{-3}	0.30	lead SNP 12
rs11081251	18	674440	A	C	1.28 (1.04-1.59)	1.75X 10^{-2}	0	0.4 (1)
rs61122623	7	87196129	T	C	7.19 (1.18-43.7)	3.21X 10^{-2}	0	lead SNP 13
rs36092077	3	183753777	G	A	1.31 (1.02-1.68)	3.48X 10^{-2}	0	lead SNP 14

Supplementary Table 4. Testing *TYMS* rs2612091, 5' VNTR and 3'UTR haplotypes for independent effects of one polymorphism

Haplotype analyses were performed in PLINK using the *--independent-effect* command, in which for each polymorphism in turn, alleles are analysed for an association with toxicity whilst keeping the genotypes of the other polymorphisms constant. The test produces a p-value for each such test and then an overall p-value for that polymorphism which shows whether that polymorphism has a consistent association with toxicity regardless of background haplotype genotype. The first three panels show the effects of varying the 5' VNTR allele, 3'UTR allele and rs2612091 allele respectively. Only rs2612091 shows a significant effect overall. The lower two panels show two-polymorphism analyses in which rs2612091 is varied whilst 5'VNTR and 3'UTR alleles are held constant. Note that some rare haplotypes are not shown.

Test SNP	5'VNTR 3'UTR rs2612091	OR for effect of each test SNP allele on haplotypes	OR for pooled effect of both test SNP alleles	p-value
5'VNTR (3 SNP model)	2R/ins/G	1 (ref)	1 (ref)	0.65
	3R/ins/G	1.04		
	2R/del/A	0.89	0.83	0.34
	3R/del/A	0.82		
	2R/ins/A	0.95	0.80	0.081
	3R/del/A	0.77		
	overall			0.17
3'UTR (3 SNP model)	2R/ins/G	1 (ref)	1 (ref)	n/a
	3R/ins/G	1.04	1.04	n/a
	2R/del/A	0.89	0.92	0.67
	2R/ins/A	0.95		
	3R/del/A	0.82	0.80	0.33
	3R/del/A	0.77		
	overall			0.61
rs2612091 (3 SNP model)	2R/ins/G	1 (ref)	1 (ref)	0.66
	2R/ins/A	0.95		
	3R/ins/G	1.04	0.84	0.00068
	3R/ins/A	0.77		
	2R/del/A	0.89	0.88	n/a
	3R/del/A	0.82	0.82	n/a
	overall			0.0021
rs2612091 (2 SNP model)	2R/G	1 (ref)	1 (ref)	0.18
	2R/A	0.92		
	3R/G	1.04	0.84	0.00051
	3R/A	0.79		
	overall			0.00053
rs2612091 (2 SNP model)	ins/G	1 (ref)	1 (ref)	
	ins/A	0.80		n/a
	del/A	0.83	0.90	n/a
	overall			1.47E-06

Supplementary Table 5. Set test analyses of capecitabine/5-FU pathway genes
In order to determine whether there was evidence in QUASAR2 of additional toxicity associations that had not reached formal statistical significance for individual SNPs or rare variants, we performed association tests based on sets of variants. The set tests used SNPs within 25kb of each of the 25 capecitabine/5-FU pathway genes plus *ENOSF1*. Prior to analysis, the known *DPYD* 2846 and *2A variants and the newly identified *DPYD* rs12132152, *DPYD* rs7548189 and *TYMS* rs2612091, as well as anything in linkage disequilibrium of $r^2>0.1$ with these SNPs (including the *TYMS* 5'VNTR and 3'UTR polymorphisms), were removed. Tests were performed by individually testing the association of each SNP under an allelic model using logistic regression adjusted for age, treatment arm and gender, permuting the outcome data and re-testing 10,000 times, then comparing the observed distribution of p-values to those from randomly assigned toxicity data for each set (i.e. per gene or across all SNPs). Using a false discovery rate of q=0.05 (p~0.005), we found no convincing evidence for additional associations at any gene or in the set of variants as a whole. We did, however, note suggestive evidence of associations between variants at the *TYMP* locus and HFS and diarrhoea.

SET	No. SNPs	Global 012v34	Global 01v2v34	HFS 012v34	HFS 01v2v34	Diarrhoea 012v34	Diarrhoea 01v2v34
ABCB1	77	0.29	1	1	1	1	1
ABCC3	64	1	1	1	1	0.55	0.49
ABCC4	221	0.86	0.87	1	1	0.91	0.75
ABCC5	100	0.33	1	1	1	0.13	0.074
ABCG2	57	0.093	1	0.37	0.60	1	1
CDA	25	1	0.24	0.38	0.20	1	0.40
CES1	24	1	0.10	0.19	0.11	1	0.16
CES2	59	0.18	0.28	0.0092	0.046	1	1
DPYD	189	0.31	0.040	0.52	0.52	0.53	0.20
DPYS	69	1	0.32	1	0.58	1	0.70
ENOSF1	22	0.11	0.27	1	0.25	1	1
MTHFR	37	1	1	1	1	1	0.50
PPAT	29	1	1	1	1	1	0.10
RRM1	29	1	1	0.17	1	1	1
RRM2	19	1	1	1	1	1	1
SLC22A7	26	1	1	1	1	0.014	0.22
SLC29A1	26	1	1	1	1	1	1
TK1	35	1	1	1	0.43	1	1
TYMP	92	1	0.24	0.035	0.025	0.055	0.029
TYMS	23	0.12	0.28	1	0.25	1	1
UCK1	43	0.13	0.16	0.25	0.36	0.013	0.0038
UCK2	21	1	1	1	1	0.063	0.31
UMPS	34	0.13	0.085	0.15	0.035	1	0.32
UPB1	30	1	1	1	1	1	1
UPP1	16	1	1	1	1	0.089	1
UPP2	42	1	0.061	0.078	0.25	1	1
**As One Set***	**1393**	**0.72**	**0.36**	**0.67**	**0.55**	**0.10**	**0.12**

*There is some overlap in the SNPs contained in the *TYMS* and *ENOSF1* set.

Supplementary Table 6. Associations between *DPYD* coding regions variants and capecitabine toxicity in QUASAR2.
The Table shows polymorphisms and rare variants present on the tagSNP or exome arrays, together with summary statistics of association with toxicity in the meta-analysis of the two arms of QUASAR2. MAF=minor allele frequency.

Variant ID						Arm A						Arm B						Overall analysis	
Chr	Position	rs#	Classical ID	Nucleotide	Amino Acid	cases_AA	cases_AB	cases_BB	controls_AA	controls_AB	controls_BB	cases_AA	cases_AB	cases_BB	controls_AA	controls_AB	controls_BB	Meta Beta	Meta P
1	97,547,947	rs67376798	2846A>T	c.T2846A	p.D949V	0	5	137	0	2	328	0	3	190	0	0	340	-2.284433	0.0010
1	97,770,920	rs1801160	*6	c.G2194A	p.V732I	123	11	0	286	19	0	170	13	0	296	20	2	0.058056	0.8277
1	97,770,920	rs1801160	*6	c.G2194A	p.V732I	130	12	0	311	19	0	178	15	0	319	19	2	0.245417	0.3453
1	97,915,614	rs3918290	*2A	c.1905+1G>A	exon skipping	140	2	0	330	0	0	192	1	0	338	2	0	1.308234	0.1793
1	97,981,343	rs55886062	*13	c.T1679G	p.I560S	141	1	0	330	0	0	192	1	0	339	1	0	0.57783	0.6975
1	97,981,395	rs1801159	*5	c.A1627G	p.I543V	89	33	12	198	95	11	116	61	6	208	98	12	0.071863	0.5601
1	97,981,421	rs1801158	*4	c.G1601A	p.S534N	135	7	0	317	13	0	183	10	0	328	12	0	0.302946	0.3688
1	98,039,419	rs56038477		c.G1236A	p.E412E	126	8	0	296	9	0	171	12	0	312	6	0	0.967362	0.0081
1	98,144,726	rs45589337		c.A775G	p.K259E	0	2	140	0	2	328	0	2	191	0	8	332	0.206601	0.7231
1	98,165,091	rs2297595		c.A496G	p.M166V	110	24	0	242	59	4	152	30	1	265	52	1	-0.141077	0.4151
1	98,348,885	rs1801265	*9A	c.C85T	p.C29R	8	40	86	18	110	177	8	59	116	21	117	180	0.204385	0.0781

Supplementary Figure 1. LD between selected variants near (a) *DPYD* and (b) *TYMS/ENOSF1* (left = D'; right=R^2).
Haplotype frequencies from Haploview EM algorithm are shown for *TYMS.*

(a) *DPYD*

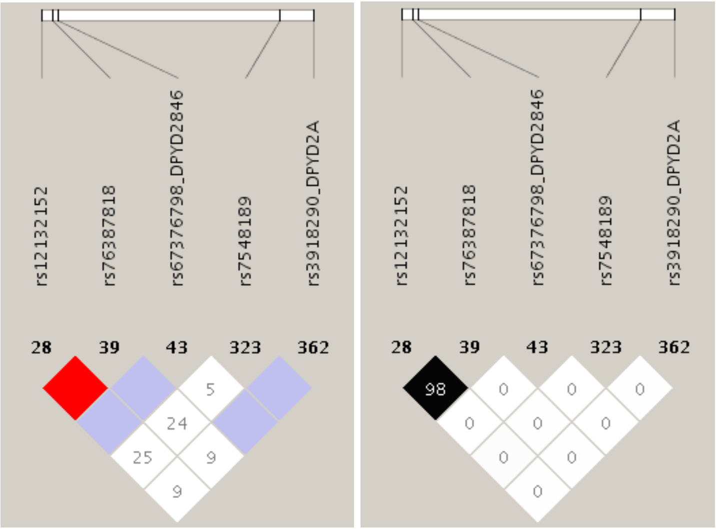

(b) *TYMS*

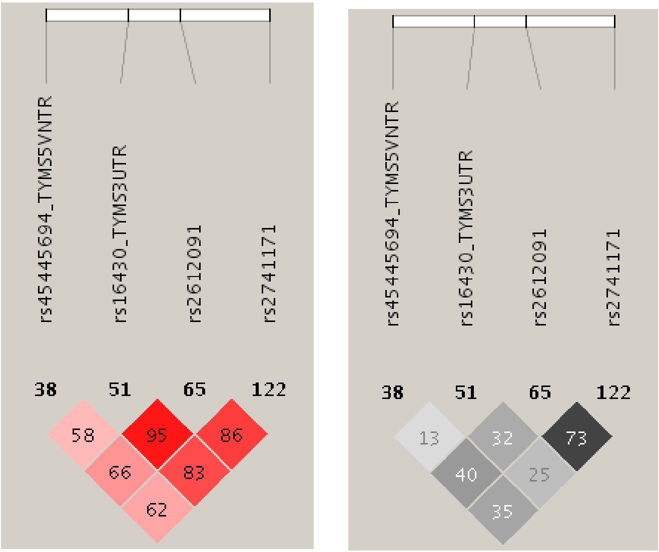

5'VNTR / 3'UTR / rs2612091		r=risk p=protective
2R/ins/G	0.366	r/r/r
3R/del/A	0.244	p/p/p
3R/ins/A	0.204	p/r/p
3R/ins/G	0.073	p/r/r
2R/del/A	0.061	r/p/p
2R/ins/A	0.045	r/r/p

Supplementary Figure 2. Sequencing coverage achieved

UCSC Bioinformatics Genome Browser user-supplied tracks showing the coverage achieved across *DPYD* (top) and *TYMS* (bottom) with Roche/454 amplicon sequencing. Top panel of each image is the coverage achieved for the 100-patient high toxicity pool; bottom panel of each image is for the 100-patient low toxicity pool. Y-axis range is from 0 coverage to over 40,000 reads per locus per pool; horizontal line on each panel marks 3,000 read coverage per pool (ie, 30x coverage per patient), achieved for all exonic loci except for some in *TYMS* exon 1 and exon 5; loci not reaching coverage goal were filtered prior to analysis.

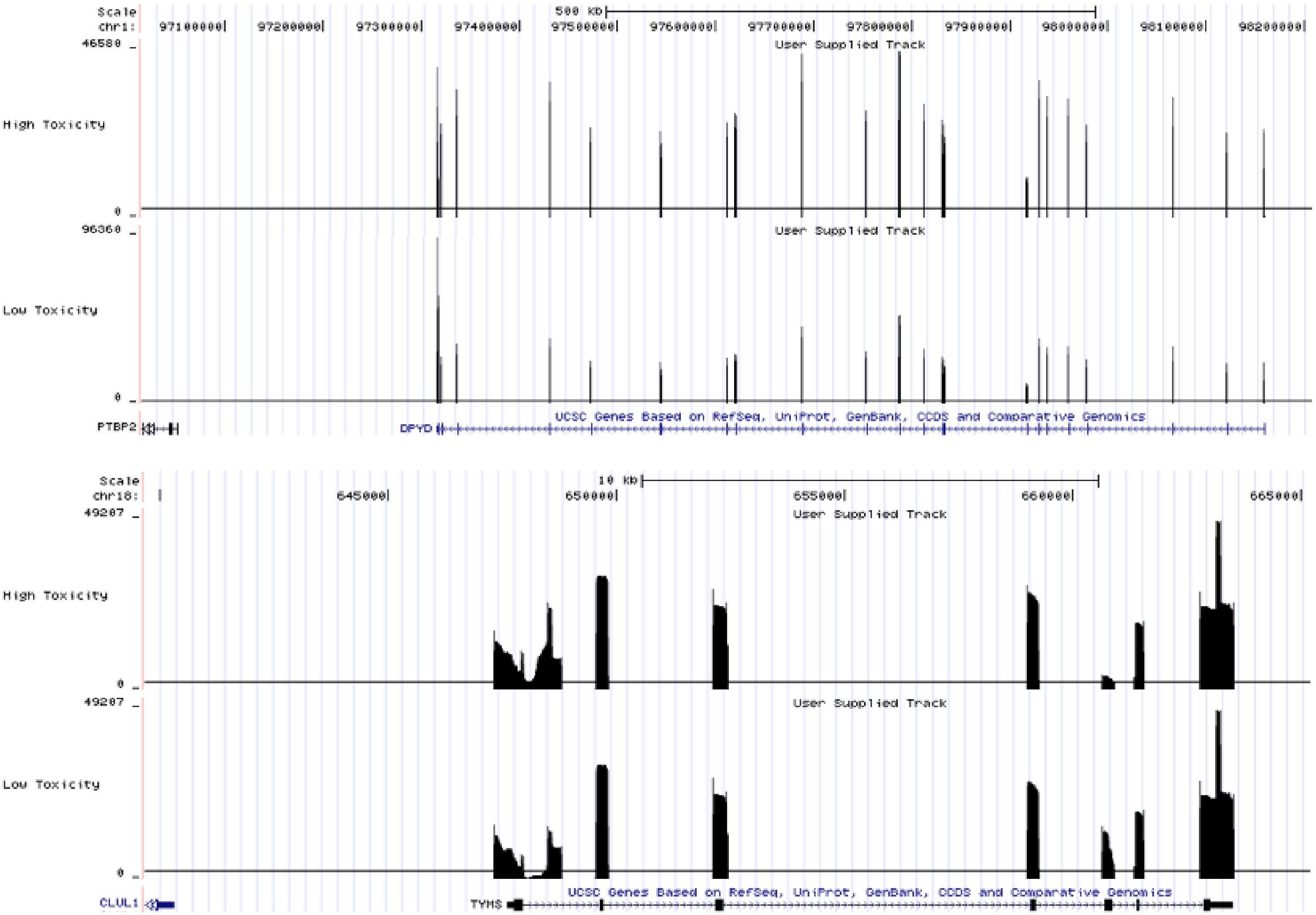

Supplementary Figure 3. Filtering of variants identified by Roche/454 sequencing of *TYMS* and *DPYD* exons

remaining variants

74 AVA-called exonic variants

↓

40 with 3,000+ reads in Fw and Rv

↓

39 with bi-directional sequencing read counts
(at least 50 reads in each direction)

↓

19 with similar allele frequency
percentages in Fw and Rv reads

↓

Remaining variants checked manually for:
- -homopolymers
- -runs of consecutive SNPs
- -early read termination
- -proximity to amplicon end
- -unusually high sequencing noise

↓

9 true variants called (all in *DPYD*)

7 dbSNPs on SNP/EXM array	1 dbSNP not on SNP/EXM array	1 novel variant
rs56038477 (1236)	rs17376848 (T1896C; F632F)	1:97981371 (G1651A; A551T)
rs1801158 (1601)		
rs1801159 (1627)		
rs1801265 (85)		
rs1801160 (2194)		
rs67376798 (2846)		
rs2297595 (496)		

Supplementary Figure 4. ROC curve

No additional independent data set was available to test the performance of a model to predict 5-FU toxicity based on the previously-reported capecitabine toxicity variants and our new data. However, in order to provide clues as to the possible clinical utility of our findings, we used the QUASAR2 data set and incorporated *DPYD* 2846T>A (rs67376798), *DPYD* *2A (rs3918290), *DPYD* rs12132152, *DPYD* rs7548189, *DPYD* p.Ala551Thr, and *TYMS* rs2612091 into a ROC analysis for prediction of global grade 012v34 capecitabine-related toxicity. 938 patients were analysed, applying a score for each patient that summed

(number of harmful alleles at each polymorphism) x (beta coefficient per allele)

The three rare *DPYD* variants were assumed to be functionally equivalent and hence combined for the purposes of this analysis into a test of any rare functional allele *versus* no rare allele (OR=7.6, p=4.5x10^{-4}). We found the area under curve (AUC) to be 0.66 (95% CI 0.63-0.70). At the cut-off for which the maximum proportion of patients were correctly classified (69%), sensitivity was 27% (95% CI 23-33%), specificity was 91% (95% CI 88-93%), positive predictive value was 60% (PPV: 95% CI 52-68%), and negative predictive value was 71% (NPV: 95% CI 68-74%) (Figure 3). Although this result must be treated cautiously given that it is derived from the same data set used for variant discovery, we note that using just the previously-reported *DPYD* 2846T>A and *2A and *TYMS* 5'VNTR and 3'UTR variants, the equivalent AUC was lower at 0.59.

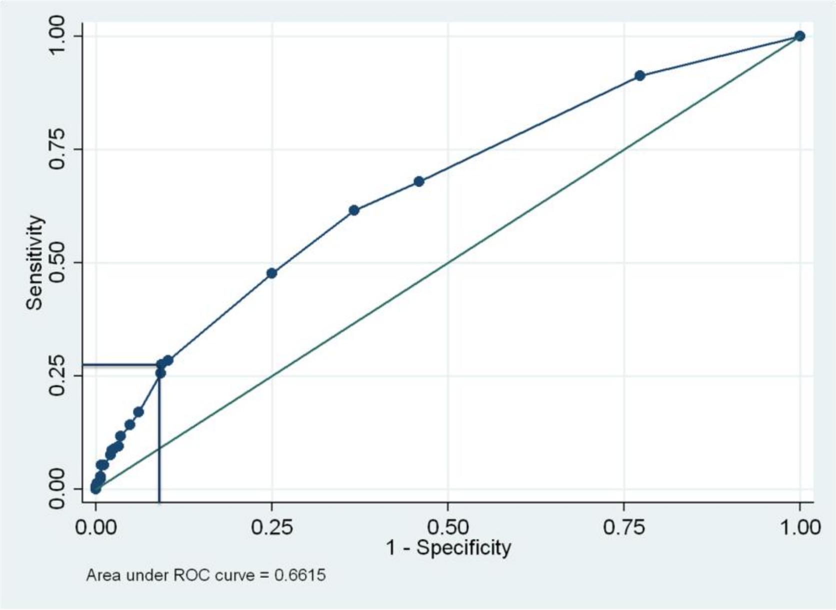

Supplementary Methods

Genotyping

DNA was extracted from buffy coat samples using conventional methods and samples with sufficient DNA and complete clinical data (N=994) were genotyped on the Hap300/370CNV, Hap610 or Omni2.5 Illumina tagging SNP arrays. 29 samples were excluded following principal component analysis as they did not cluster with CEU HapMap3 samples, 12 samples were excluded because per sample call rates were < 95% and 7 samples were excluded because of gender discrepancies. Quality control procedures were performed to eliminate poorly-performing polymorphisms, as described in [1]. After applying quality control procedures QUASAR 2 patient genotypes were available for SNPs present on the Illumina Hap300/370CNV (N=484), Hap610 (N=364) or Omni2.5 (N=92) tagging SNP arrays. Data were also available for a largely overlapping set of 968 QUASAR2 patients genotyped on the Illumina HumanExome12v1_A or -12v1-1_A arrays, which were designed to capture uncommon protein-coding variation [2]. The additional samples genotyped on the exome arrays but not tagging SNP arrays had missing DNA or clinical data at time of genotyping using tagging arrays. Base calling for all platforms was performed using Illumina Genome Studio and, for exome arrays, additionally by Z-Caller [3], applying a z-score of 7 based on the concordance of calls with Illumina Genome Studio for common variants (99.3%). 8,694 polymorphisms were present on both the SNP tagging arrays and exome array and there was 99.2% genotyping concordance for these SNPs.

For each of the 25 capecitabine/5-FU pathway genes (Supplementary Table 1), we identified genetic variants that were present on one or more of the Hap300/370, Hap610 or exome arrays and that lay within 25kb of the coding region of one of the genes. We used imputation to obtain missing genotypes arising from differences in array content: haplotypes were phased using SHAPEITv2 [4] and imputation performed using IMPUTEv2 [5], employing a 250kb buffer region and the 1000 genomes August 2012 release (all ethnicities) as a reference panel. Only SNPs with an IMPUTEv2 info score of at least 0.95 on each array individually were taken into further analysis by SNPTESTv2 [6]. Further exclusion criteria were a SNPTEST info score below 0.95 on the

pooled score from the three SNP arrays, a minor allele frequency below 0.01 and a Hardy-Weinberg equilibrium p-value below 0.0001. Genotyping and imputation provided a total of 1,456 genetic variants for analysis.

The accuracy of imputation was further tested for specific SNPs using constitutional genotypes from 190 white UK individuals who had been whole genome-sequenced using the Complete Genomics platform. The input for IMPUTE2 was genotype files that represented the SNP content of the arrays used to genotype QUASAR2. The imputed SNP genotype probabilities were converted into genotypes using gtool only if the probability of a particular genotype was ≥0.9. Real genotypes and imputed ones were then compared to determine concordance and missingness.

Further genotyping was performed for the *TYMS* 5'VNTR and 3'UTR variants by previously-described methods [7,8]. Additional genotyping of the *DPYD* 2846T>A and *DPYD* *2A variants was performed by allele-specific amplification by KASPar [9] for the small number of patients not genotyped using the exome arrays.

For loci at which significant or borderline significant associations between genetic variants and toxicity were detected, we performed fine mapping studies by using the methods above to impute all SNPs in a 1.5Mb flanking region, in order to refine the association signal.

454 Sequencing

Sequencing of the coding regions of *DPYD* and *TYMS* was performed by Roche/454 Titanium GS FLX technology according to the specified amplicon sequencing protocol (see http://454.com/downloads/my454/documentation/gs-junior/method-manuals/GSJunior_AmpliconLibraryPrep-RevJune2010.pdf; further details available from authors). Specifically, we selected the 100 patients with the highest levels of 5-FU-related toxicity ("HiTox"), specifically grade 3 or grade 4 diarrhoea in the first 4 cycles of treatment and or other grade 3/4 toxicities in the first 4 cycles of treatment. We also selected 100 patients with no adverse toxicity events during the entire duration of

treatment (“LoTox”). We used primer3 to design PCR primers and reactions to cover all 23 *DPYD* exons (27 amplicons; 4,784bp) and 7 *TYMS* exons (9 amplicons; 2,276bp) (primers and conditions available upon request). Constitutional DNA samples from each patient were quantitated using PicoGreen, diluted to equal measured concentrations and formed into 10 pools of 20 patients each. Pools were then PCR-amplified for each of the 36 amplicons. Missing or undesired amplicons were identified by an Agilent High Sensitivity DNA Kit. Successful amplicons were quantified by PicoGreen according to the 454 protocol, equalised in concentration and formed into one 100-patient HiTox pool and one 100-patient LoTox pool for sequencing. We aimed to achieve a minimum read depth of 3,000 per target locus per pool (that is about 30x coverage per patient).

Mapping and initial variant calling were performed by Roche/454 software (GS Mapper and AVA). Variants were then filtered to include only those with 3,000+ reads in total, at least 50 reads in each direction and similar allele frequencies in the forward and reverse directions. Variants were then removed if they fell within a homopolymer, a run of consecutive SNPs, an early-terminating read, the end of a full length read, or an area of evidently poor sequence quality. Variant frequencies were determined, per pool, as the proportion of total reads (forward plus reverse) containing the minor allele. Within our targets, we confirmed the presence and allele frequency of known SNPs using our array data. The novel variants were validated with Sanger sequencing of the individual patients who comprised the pool (details available on request).

Functional annotation of variants was performed with ANNOVAR. mRNA expression data were obtained from Genevar [10] and from The Cancer Genome Atlas (TCGA). We analysed these data according to the methods of Li et al [11].

Putative associations with toxicity were determined according to the estimated number of variant and wildtype reads present in the HiTox and LoTox pools (Pearson’s Chi Squared or Fisher’s exact test).

Supplementary References

1. Dunlop MG, Dobbins SE, Farrington SM*, et al.* Common variation near CDKN1A, POLD3 and SHROOM2 influences colorectal cancer risk. Nat Genet 2012; **44**: 770-6.
2. Huyghe JR, Jackson AU, Fogarty MP*, et al.* Exome array analysis identifies new loci and low-frequency variants influencing insulin processing and secretion. Nat Genet 2013; **45**: 197-201.
3. Goldstein JI, Crenshaw A, Carey J*, et al.* zCall: a rare variant caller for array-based genotyping: genetics and population analysis. Bioinformatics 2012; **28**: 2543-5.
4. Delaneau O, Howie B, Cox AJ*, et al.* Haplotype estimation using sequencing reads. Am J Hum Genet 2013; **93**: 687-96.
5. Howie B, Fuchsberger C, Stephens M*, et al.* Fast and accurate genotype imputation in genome-wide association studies through pre-phasing. Nat Genet 2012; **44**: 955-9.
6. Marchini J, Howie B, Myers S*, et al.* A new multipoint method for genome-wide association studies by imputation of genotypes. Nat Genet 2007; **39**: 906-13.
7. Dotor E, Cuatrecases M, Martinez-Iniesta M*, et al.* Tumor thymidylate synthase 1494del6 genotype as a prognostic factor in colorectal cancer patients receiving fluorouracil-based adjuvant treatment. J Clin Oncol 2006; **24**: 1603-11.
8. Horie N, Aiba H, Oguro K*, et al.* Functional analysis and DNA polymorphism of the tandemly repeated sequences in the 5'-terminal regulatory region of the human gene for thymidylate synthase. Cell Struct Funct 1995; **20**: 191-7.
9. Cuppen E. Genotyping by Allele-Specific Amplification (KASPar). CSH Protoc 2007; **2007**: pdb prot4841.
10. Yang TP, Beazley C, Montgomery SB*, et al.* Genevar: a database and Java application for the analysis and visualization of SNP-gene associations in eQTL studies. Bioinformatics 2010; **26**: 2474-6.
11. Li Q, Seo JH, Stranger B*, et al.* Integrative eQTL-based analyses reveal the biology of breast cancer risk loci. Cell 2013; **152**: 633-41.
